# Systemically Silencing Long Non-coding RNAs Maclpil With Short Interfering RNA Nanoparticles Alleviates Experimental Ischemic Stroke by Promoting Macrophage Apoptosis and Anti-inflammatory Activation

**DOI:** 10.3389/fcvm.2022.876087

**Published:** 2022-05-06

**Authors:** Yan Wang, Cuiying Liu, Yong Chen, Tiffany Chen, Tao Han, Lixiang Xue, Baohui Xu

**Affiliations:** ^1^Institute of Medical Innovation and Research, Peking University Third Hospital, Beijing, China; ^2^Medical Research Center, Peking University Third Hospital, Beijing, China; ^3^School of Nursing, Capital Medical University, Beijing, China; ^4^Department of Neurology, Peking University Third Hospital, Beijing, China; ^5^Department of Surgery, Division of Vascular Surgery, Stanford University School of Medicine, Stanford, CA, United States

**Keywords:** ischemic stroke, neuroinflammation, macrophages, lncRNA maclpil, SiRNA nanoparticles

## Abstract

**Background:**

Maclpil is a proinflammatory long non-coding RNA highly expressed on monocyte-derived macrophages in the ischemic brain. This study investigated the impact and the mechanisms of systemically delivering nanoparticle Maclpil short interfering RNA (siRNA) on experimental ischemic stroke in a mouse model.

**Methods:**

Ischemic stroke (focal cerebral ischemia) was induced in male C57BL/6 mice through the middle cerebral artery occlusion. Three hours thereafter, mice were intravenously injected with Maclpil siRNA or scramble siRNA nanoparticles. Bone marrow cell-derived macrophages were transfected with Maclpil or scramble siRNA and subjected to oxygen glucose deprivation culture. The influence of silencing Maclpil on stroke outcomes, neuroinflammation, and macrophage fates was assessed *via* histology, flow cytometry, Western blotting, and quantitative PCR analysis.

**Results:**

Three days following stroke induction, siRNA silencing Maclpil substantially reduced ischemic infarction size and improved neurological behaviors. Silencing Maclpil also markedly attenuated the accumulation of monocyte-derived macrophages, CD4^+^ T cells, and CD8^+^ T cells in the ischemic hemisphere without affecting microglia cellularity. Reciprocally, myeloid cells and both subsets of T cells were elevated in mouse peripheral blood following Maclpil siRNA treatment. Under oxygen glucose deprivation conditions that mimicked hypoxia and hypoglycemia *in vitro*, Maclpil siRNA silencing augmented macrophage apoptosis in conjunction with upregulation of proapoptotic Bax and caspase 3 expressions. siRNA knocking down Maclpil skewed macrophages from proinflammatory classical toward anti-inflammatory alternative activation as evidenced by increased arginase 1, Ym1, and Fizz1 and reduced inducible nitric oxide synthase, IL-1β, and TNF-α mRNA levels. Consistent with macrophage phenotype switching, silencing Maclpil by siRNA enhanced fatty acid oxidation as indicated by increased mRNA levels of 3 key metabolic enzymes (ACADM, ACADVL, and HADHA).

**Conclusion:**

Systemically silencing Maclpil by siRNA nanoparticles attenuated experimental ischemic stroke by promoting macrophage apoptosis and anti-inflammatory alternative activation. Identifying and targeting Maclpil human homolog(s) may help develop a novel therapy for stroke clinical management.

## Introduction

Stroke-related death has increased by 26% from 1990 to 2010 and has affected approximately 800,000 people in the United States in 2020 (1, 2). In China, ischemic stroke is predominant and is the first leading cause of mortality (3). Currently, tissue plasminogen activator is the only drug for managing acute ischemic stroke approved by United States Food and Drug Administration with a narrow therapeutic time window. Thus, it is urgent to develop alternative effective pharmacological therapies for stroke treatment.

Stroke-induced neuroinflammation has attracted more attention in stroke management, particularly immune intervention (4). Following ischemia onsets, the blood-brain barrier is disrupted and circulating leukocytes are recruited into the inflamed brain. Using mass spectrometry, we previously found that monocytes-derived macrophages were the predominant leukocytes that infiltrated into the ischemic hemisphere during the acute phase of the stroke (5).

Long non-coding RNAs (lncRNAs) are untranslated regulatory RNAs with more than 200 nt and are important in multi-pathophysiological processes including stroke pathogenesis. We and others have shown that certain lncRNAs were highly expressed in the experimental ischemic brain (6–8) with specific upregulation of Gm15428 in monocyte-derived macrophages (9–11). In bone marrow-derived macrophages (BMDCs), silencing Gm15428 significantly reduced the expression of lymphocyte cytosolic protein 1 (LCP1) and inhibited pro-inflammatory macrophage polarization. Thus, Gm15428 was named as *ma*crophage *c*ontained *L*CP1 related *p*ro-inflammatory lncRNA or Maclpil (12). Further, adoptively transferring classically activated and Maclpil-silenced macrophages attenuated ischemic infarct size, neuroinflammation, neurological defects, and proinflammatory mediator expression, suggesting Maclpil as a proinflammatory IncRNA (12). However, it remains unknown whether systemically inhibiting Maclpil influences ischemic stroke and its associated inflammation.

This study utilized nanoparticle-conjugated short interfering RNA (siRNA) to systemically inhibit IncRNA Maclpil following ischemic stroke induction and to evaluate its impact on stroke and neuroinflammation. Oxygen glucose deprivation (OGD) assay that simulates hypoglycemia and hypoxia in stroke was used to explore potential mechanisms, particularly the fate and activity of macrophages.

## Materials and Methods

### Animals

Male C57BL/6 mice, weight 22–25 g aged 8–10 weeks were purchased from and housed at Peking University or Capital Medical University under a 12:12 h light–dark cycle with free access to food and water. All animal experiments were performed in consistence with the animal research reporting *in vivo* experiments guidelines and approved by the Peking University and Capital Medical University Animal Care and Use Committees.

### Focal Cerebral Ischemia

Focal cerebral ischemia was induced by a 45 min transient middle cerebral artery occlusion (MCAO) procedure by inserting a silicone-coated 6–0 monofilament (Doccol Corp., Sharon, MA, United States) into the left common carotid artery to block the MCA as detailed previously (13). Anesthesia was induced and maintained with 5% and 2% isoflurane throughout the surgery. Body temperature was monitored and maintained at 37 ± 0.5°C using a surface heating pad. Sham-operated mice underwent the same procedures without monofilament insertion.

### Neurological Behavior Tests

Neurobehavioral tests were conducted using a modified neurological severity score (mNSS) system to comprehensively assess neurological functions from four different aspects, including motor, sensory, balance, and reflex tests (14). The total mNSS scores range from 0 to 14, in which 0 represents normal and 14 represents the highest degree of neurological defects. For the motor assay, after raising the mouse by the tail, the bend and torsion of the limbs were graded as the score 0–3. Walking posture was also graded as the score 0–3. For balance test, mice were placed on a beam to observe whether the mice were able to maintain their balance to keep walking on or fell off the beam and were scored from 0 to 6. In sensory and reflex tests, pinna and corneal reflexes were examined, respectively, scored from 0 to 2. All these tests were carried out by an investigator who was blinded to experiment group assignment.

### Infarction Size Measurement

Three days following MCAO surgery, mice were euthanized *via* overdose isoflurane inhalation. Brains were harvested, sliced into five slices (2 mm thickness), fixed in 4% paraformaldehyde, and stained in 2% 2, 3, 5-triphenyltetrazolium chloride (TTC, Cat^#^ T8877, Sigma–Aldrich, St. Louis, MO, United States) at 37°C overnight. Infarction size was measured in scanned slide images using ImageJ software and calculated as the percentage of area relative to the non-ischemic hemisphere (15).

### Tissue Immunofluorescence Staining

The frozen brain was sectioned (20 μm thickness). Sections were fixed with 4% paraformaldehyde and stained with a rat anti-mouse CD68 antibody (Cat^#^ ab955, Abcam, Waltham, MA, United States) or rabbit anti-mouse iNOS (Cat^#^ ab15323, Abcam) (all 1:100) followed by incubation with Alexa 488-conjugated goat anti-rat antibody (Cat^#^ A-11001) or Alexa 488-conjugated goat anti-rabbit antibody (Cat^#^ A11008) (All from Invitrogen, Waltham, MA, United States). Sections were counterstained with DAPI and mounted for imaging acquisition.

### Preparation and Treatment of Bone Marrow-Derived Macrophages

Bone marrow-derived macrophages were generated by differentiating C57BL/6 mouse bone marrow cells in the presence of recombinant mouse macrophage colony stimulation factor (M-CSF, 10 ng/ml, Cat^#^ PMC2044, Thermo Fisher Scientific, Waltham, MA, United States). To induce OGD, macrophages were suspended in deoxygenated and glucose-free DMEM media. Cells were then incubated in an oxygen deprivation chamber at 1% O_2_, 5% CO_2_, and 95% N_2_ and 37°C. Following OGD, cells were transferred to normal media and incubated at 5% CO_2_ at 37°C for re-oxygen and re-glucose treatment overnight (13).

### Flow Cytometric Analysis

The ischemic hemisphere was harvested, minced, and suspended in RPMI-1640 media and filtered through a 70 μm cell strainer (12). Two milliliters of 70% Percoll (Cat^#^17089019, Cytivia, O’Fallon, MO, United States) were loaded to the bottom of the cell suspension and centrifuged at 600 *g* for 30 min. Cells at the interphase were collected and re-suspended in phosphate-buffered saline (PBS). For the blood sample, the peripheral mononuclear cells were isolated using the Ficoll reagent (Cat^#^17-1440-02, GE Healthcare, Sweden). Cells were stained with antibodies against CD45-BV510, CD11b-FITC, CD4-APC-Cy7, and CD8-APC for 30 min at 4°C followed by incubation with 7-AAD for 5 min, washed twice with PBS, and resuspended in 100 μl PBS. All antibodies were purchased from BioLegend Inc., San Diego, CA, United States. The stained cells were analyzed on the Beckman CytoFLEX S flow cytometer (Beckman Coulter, Indianapolis, IN, United States).

### Preparation and Systemic Delivery of Short Interfering RNA Nanoparticles

siRNA or scramble siRNA was conjugated to nanoparticles using versatile DNA/siRNA transfection reagent following the manufacturer’s instructions (Cat# 114-15, jetPRIME™, Polyplus-Transfection SA, New York, NY, United States). Briefly, 110 picomoles of siRNA were sequentially mixed with 200 μl jetPRIMETM buffer and 4 μl jetPRIME TM reagent and incubated for 15 min at room temperature for immediate use. For *in vivo* siRNA delivery, *in vivo*—jetPEI ^®^ (Reference number 101000040) was used following the instruction, and siRNA usage was 1 mg/kg.

### Apoptosis Assay

Cells were stained using FITC Annexin V (Cat^#^ 640905, Biolegend), counterstained with propidium iodide (Cat^#^ P8080, Solarbio, China), and analyzed on the Beckman CytoFLEX S flow cytometer. Apoptotic cells were defined as Annexin^+^PI^+^ cells.

### Western Blot

Cells were lysed in RIPA buffer and subjected to Western -blotting analysis. Reagents for Western -blotting were anti-Bax antibody (1:1000, Cat^#^ 60267-1, Proteintech, Wuhan, China), anti-caspase 3 antibody (1:1500, Cat^#^ 66470-2, Proteintech), anti-GAPDH antibody (1:2000, Cat^#^ab181603, Abcam), and anti-rabbit IgG antibody conjugated to HRP (1:2000, Cat^#^ZB-2305, zsbio, Beijing, China). The protein marker was purchased from Applygen, Beijing, China (10 KDa-180KDa, Cat^#^ P1103).

### Digital Droplet Quantitative Reverse Transactional PCR Assay

Total RNA was extracted using the total RNA kit (Cat^#^DP419, TIANGEN Biotech, Beijing, China) and transcribed into cDNA using GoScriptTM Reverse transcription system (Cat^#^ A5000, Promega, Madison, WI, United States). Sequences for gene-specific PCR primers are summarized in Table 1. Digital PCR was performed using the Sniper DQ24 digital PCR system (Sniper, Suzhou, Jiangsu, China), and gene expression levels were expressed as copy number/μl.

**TABLE 1:** Primer sequences.

Gene	Sequence (5′-3′)
Mouse Arg1 forward	AACACGGCAGTGGCTTTAACC
Mouse Arg1 reverse	GGTTTTCATGTGGCGCATTC
Mouse Ym-1 forward	TCCAGCTAACTATCCCTCCACTGT
Mouse Ym-1 reverse	GGCCCATCTGTTCATAGTCTTGA
Mouse Fizz-1 forward	CTGCCCTGCTGGGATGACT
Mouse Fizz-1 reverse	CATCATATCAAAGCTGGGTTCTCC
Mouse iNOS forward	CGAAACGCTTCACTTCCAA
Mouse iNOS reverse	TGAGCCTATATTGCTGTGGCT
Mouse TNFα forward	GAGTGACAAGCCTGTAGCC
Mouse TNFα reverse	CTCCTGGTATGAGATAGCAAA
Mouse IL-1β forward	CCAGCTTCAAATCTCACAGCAG
Mouse IL-1β reverse	GGCGTATCAGTGGGGGTCAG

### Measurements of Fatty Acid Oxidation Enzymes

Three fatty acid oxidation enzymes were analyzed to assay fatty acid oxidation using a commercial kit (Cat^#^118183, Abcam). Macrophages were transfected with Maclpil siRNA or scramble siRNA nanoparticles, underwent OGD, and harvested. Following fixation and permeabilization, cells were stained with an antibody against ACADM (Acyl-Coenzyme A dehydrogenase, C-4 to C-12 straight chain), ACADVL (ACAD Very long-chain specific acyl-CoA dehydrogenase), or HADHA (hydroxyacyl-CoA dehydrogenase/3-ketoacyl-CoA thiolase/enoyl-CoA hydratase) for 1 h followed by incubation with FITC-conjugated secondary antibody for another 1 h. The expression of individual enzymes on macrophages was analyzed on the Beckman CytoFLEX S flow cytometer and presented as mean fluorescence intensity.

### Statistical Analysis

Two-tailed student’s *t*-test were performed using GraphPad Prism. *p*-Value <0.05 was considered statistically significant.

## Results

### Systemically Silencing Long Non-coding RNA Maclpil Reduces Infarction Size and Attenuates Neurological Defects in Mice Following Ischemia

**FIGURE 1:**
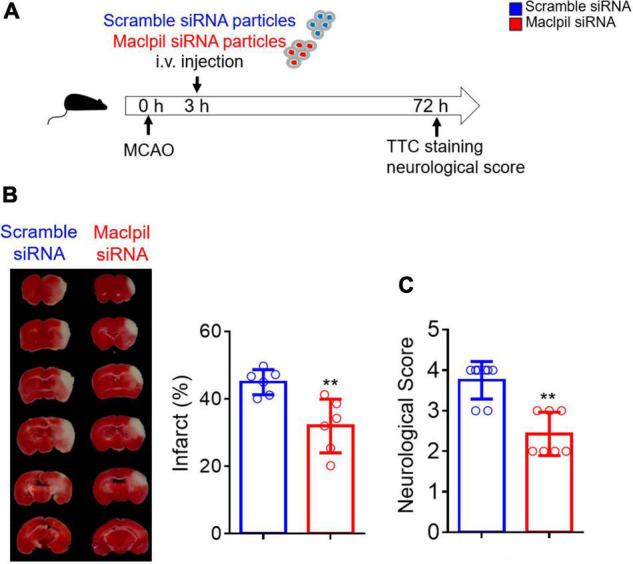
siRNA silencing Maclpil reduces ischemic infraction size and improves neurological behaviors. **(A)** Three hours after MACO surgery, mice were intravenously injected with nanoparticle conjugated Maclpil or scramble siRNA. Neurological behaviors and infarction size were assessed 3 days after the MCAO surgery. **(B)** Images for infarction area identified by TTC staining and quantification of infarction size. **(C)** Quantification of neurological score. All data are mean and SD from 6 **(B)** and 7–8 **(C)** mice in each group. Student’s *t*-test, **p < 0.01 compared to scramble siRNA treatment.

Mice were intravenously injected with siRNA nanoparticles 3 h after MCAO (Figure 1A), as our previous study showed that the number of peripheral monocytes significantly increased from 3 h after ischemia (5). The ischemic infarction and neurological score were analyzed 3 days thereafter. Infarction was significantly small in mice receiving Maclpil (30%) as compared to mice receiving scramble siRNA particles (40%) (Figure 1B). Neurological scores averaged 2.4 in mice receiving Maclpil siRNA particles as compared to mice receiving scramble siRNA particles (3.8) (Figure 1C). Thus, suppressing Maclpil mitigated ischemic infarction and neurobehavior defects.

### Maclpil Short Interfering RNA Nanoparticles Inhibit Stroke-Induced Brain Inflammation

**FIGURE 2:**
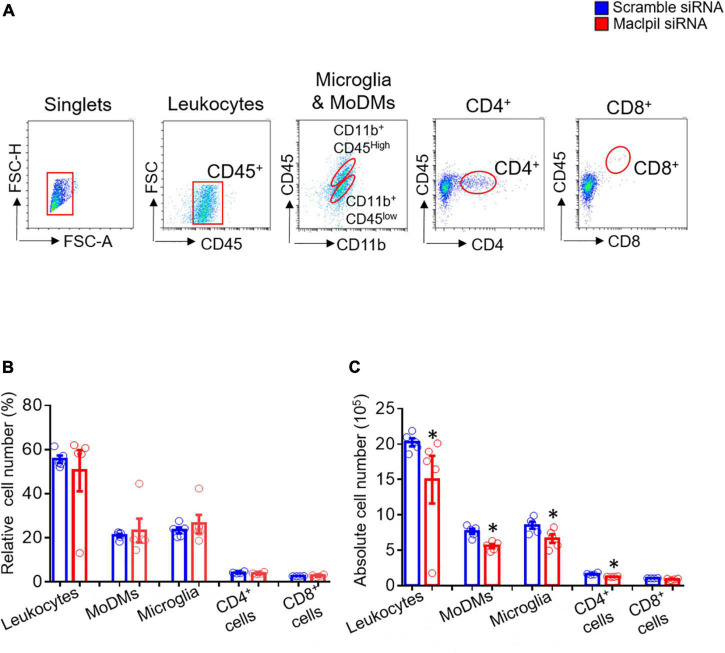
siRNA silencing Maclpil alleviates leukocyte accumulation in experimental ischemic stroke. **(A)** Flow cytometric analysis strategies for identifying individual subsets of leukocytes. Leukocytes: CD45^+^; Microglia: CD11b^+^CD45^low^; monocyte-derived macrophages (MoDMs): CD11b^+^CD45^high^; CD4^+^ cells: CD4^+^; and CD8^+^ cells: CD8^+^. **(B,C)** Quantification of relative and absolute numbers of leukocytes, MoDM, microglia, CD4^+^ cells and CD8^+^ cells. All data are given as mean and SD from 5 mice per group. Student’s *t*-test, *p < 0.05 compared to scramble siRNA treatment.

**FIGURE 3:**
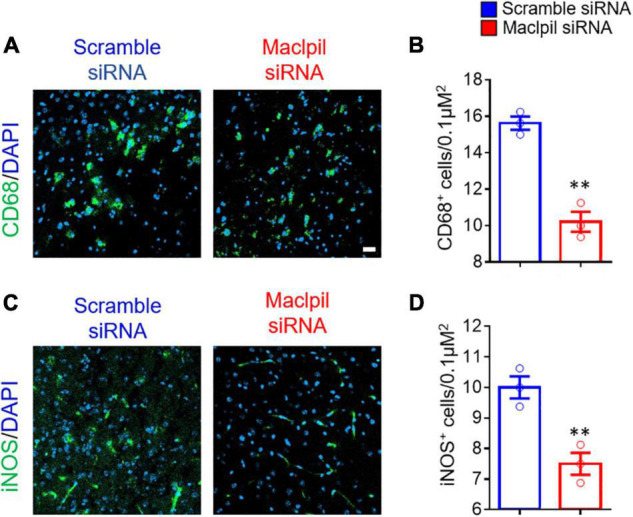
siRNA silencing Maclpil reduces cells expressing CD68 and iNOS in ischemic infraction area. Brain sections were stained with antibodies against CD68 and iNOS, respectively, counterstained with DAPI for nuclei, and imaged on a confocal microscope. **(A,B)** Representative staining images for CD68 **(A)** and CD68^+^ cell density estimated as positively stained cells/0.1 μm^2^ **(B)**. **(C,D)** Representative staining images for iNOS **(C)** and iNOS^+^ cell density quantified as positively stained cells/0.1 μm^2^ **(D)**. All data are presented as mean and SD from 3 mice in each group. Student’s *t*-test, ^**^p < 0.01 compared to scramble siRNA treatment.

**FIGURE 4:**
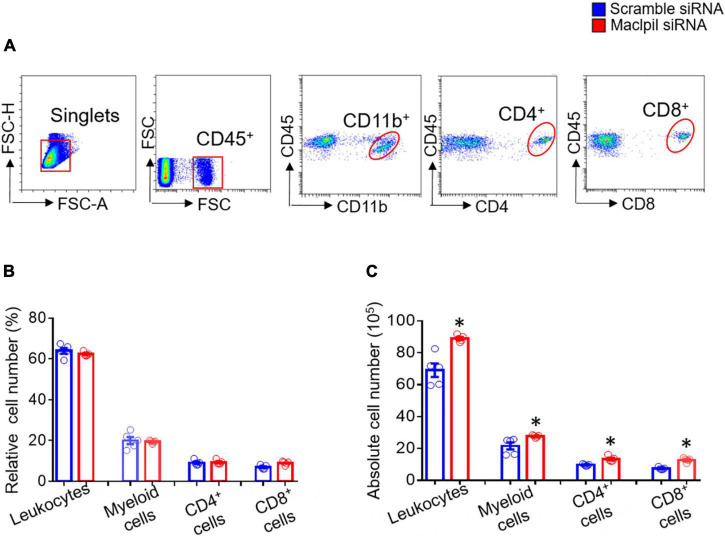
siRNA silencing Maclpil increases circulating leukocytes in experimental ischemic stroke. **(A)** Flow cytometric analysis strategies for identifying individual subsets of leukocytes. Leukocytes: CD45^+^; myeloid cells: CD45^+^CD11b^+^CD45^low^; CD4^+^ cells: CD4^+^; and CD8^+^ cells: CD8^+^. **(B,C)** Quantification of relative **(B)** and absolute **(C)** numbers of leukocytes, myeloid cells, CD4 + cells and CD8 cells. All data are given as mean and SD from 5 mice per group. Student’s *t*-test, *p < 0.05 compared to scramble siRNA treatment.

To assess the influence of Maclpil siRNA particle treatment on neuroinflammation following ischemia, we analyzed leukocytes in the ischemic hemisphere and peripheral blood using flow cytometric analysis. In the ischemic hemisphere, mice receiving Maclpil siRNA particles had less CD45^+^ cells (leukocytes and microglia) and monocytes-derived macrophages (MoDMs, CD45^high^CD11b^+^) as compared to mice receiving scramble siRNA particles (Figure 2A). These results indicate that systemically inhibiting lncRNA Maclpil alleviated neuroinflammation. Specifically, all CD45^+^ cells (leukocytes and microglia) were reduced in Maclpil siRNA (two million), as compared to scramble (one and a half mission) siRNA treated mice. Monocytes-derived macrophages (MoDM) were reduced to 5 million in Maclpil siRNA, as compared to scramble siRNA (seven and a half million) treated mouse ischemic hemisphere (Figures 2B,C). In tissue immunofluorescent staining, the densities for macrophages (CD68^+^) and iNOS^+^ cells (mostly expressed by inflammatory macrophages) were significantly reduced in the ischemic hemisphere from mice treated with Maclpil siRNA as compared to scramble siRNA treatment (Figures 3A–D). Conversely, circulating leukocytes (CD45^+^ cells) were increased in Maclpil siRNA (8.3 × 10^6^), as compared to scramble siRNA (6.8 × 10^6^) in treated mice (Figure 4A). This was also the case for CD11b^+^ myeloid cells, CD4^+^T cells, and CD8^+^T cells (Figures 4B,C). These results indicate that inhibiting Maclpil attenuated leukocyte accumulation in the ischemic brain in association with reciprocal increased retention in peripheral blood.

### Silencing Maclpil Promotes Macrophage Apoptosis After Oxygen-Glucose Deprivation Treatment

**FIGURE 5:**
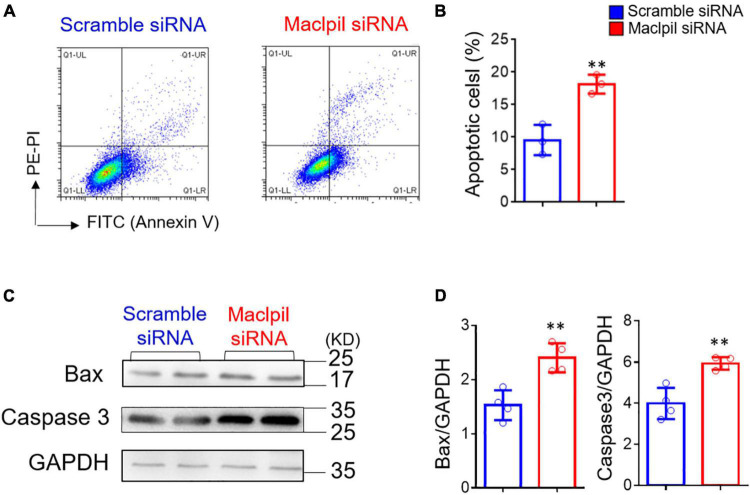
siRNA silencing Maclpil increases apoptosis and proapoptotic protein expression in macrophages undergoing oxygen glucose deprivation treatment. **(A)** Representative flow cytometric pseudocolor plots showing Annexin^+^PI^+^ apoptotic cells in Maclpil or scramble siRNA-treated bone marrow-derived macrophages (BMDCs). **(B)** Quantification of apoptotic cells in Maclpil and scramble siRNA-treated BMDCs. **(C)** Representative Western blots for proapoptotic proteins (Bax and caspase 3) and house-keeping gene (GAPDH) in Maclpil- and scramble siRNA-treated BMDCs. **(D)** Quantification of the expression of Bax and caspase 3 in Maclpil and scramble siRNA-treated BMDCs as the ratio to GAPDH. All data are mean and SD from triplicate experiments. Student’s *t*-test, ^**^p < 0.01 compared to scramble siRNA treatment.

Ischemic stroke occludes the cerebral artery, reduces blood supply, and consequently leads to oxygen and glucose deprivation (16). Thus, we used BMDMs and *in vitro* OGD assay to mimic oxygen and glucose deprivation in ischemic stroke to evaluate the influence of OGD treatment on macrophage apoptosis (Figure 5A). Annexin V and PI staining revealed increased apoptosis in BMDMs transfected with Maclpil as compared to scramble siRNA treatment (Figure 5B). In Western blotting analysis, Bax and Caspase 3, two proapoptotic molecules were significantly upregulated in Maclpil siRNA transfected BMDMs as compared to scramble siRNA transfection (Figures 5C,D). These results suggest that lncRNA Maclpil may inhibit macrophage apoptosis.

### Silencing Maclpil Polarizes Macrophages Toward an Anti-inflammatory Phenotype Under Oxygen-Glucose Deprivation Condition

**FIGURE 6:**
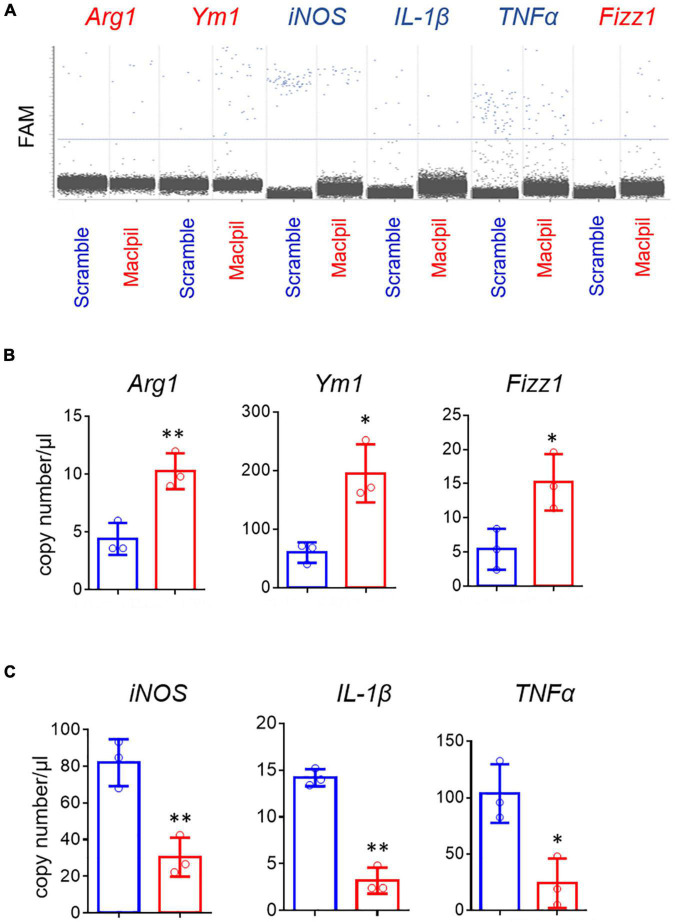
siRNA silencing Maclpil promotes anti-inflammatory gene expression in macrophages undergoing oxygen glucose deprivation treatment. Following oxygen glucose deprivation treatment, total RNA was extracted from marrow-derived macrophages (BMDCs) and subjected to reverse transcript and digital PCR amplification sequentially. **(A)** Representative 2-D fluorescence amplitude (FAM) plots acquired *via* digital PCR for individual genes in each treatment group. **(B,C)** Quantification of anti-inflammatory (Arg1, Fizz1, Ym1) gene **(B)** and pro-inflammatory (iNOS, IL-1β and TNFα) gene **(C)** expression in BMDCs from different treatment groups. All data are mean and SD from triplicate experiments. Student’s *t*-test, *p < 0.05 and ^**^p < 0.01 compared to scramble siRNA treatment.

**FIGURE 7:**
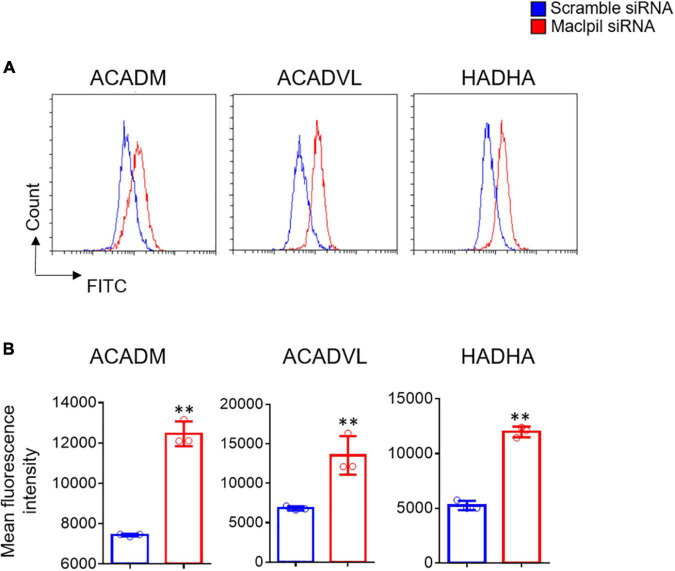
siRNA silencing Maclpil increases the expression of key fatty acid oxidation enzymes in macrophages undergoing oxygen glucose deprivation treatment. Following oxygen glucose deprivation treatment, marrow-derived macrophages (BMDCs) were stained with unlabeled primary antibodies against ACADM, ACADVL, and HADHA followed by incubation with fluorochrome-conjugated secondary antibodies and analyzed on a flow cytometer. **(A)** Representative flow cytometric overlay histograms showing the staining for ACADM, ACADVL, and HADHA in Maclpil and scramble siRNA-treated BMDCs after oxygen glucose deprivation. **(B)** Quantification of the expression levels (mean fluorescence intensity, mean and SD, *n* = 3) of ACADM, ACADVL, and HADHA in differentially treated BMDCs. Student’s *t*-test, ^**^p < 0.01 compared to scramble siRNA treatment.

In OGD experiments, scramble siRNA-transfected macrophages displayed a proinflammatory phenotype as evidenced by high levels of iNOS, IL-1β, and TNF-α mRNAs in the absence of exogenous stimuli such as LPS or IFN-γ. In contrast, macrophages switched toward an anti-inflammatory phenotype with increased mRNA levels for Arg1, Ym1, and Fizz 1 and reduced mRNA levels for iNOS, IL-1β, and TNF-α in digital droplet PCR assay (Figure 6). Consistent with macrophage switch and the well-documented role of fatty acid oxidation in anti-inflammatory macrophage activation (17), the mRNA levels for 3 fatty acid oxidation enzymes (ACADM, ACADVL, and HADHA) were substantially increased in macrophages treated with Maclpil as compared to scramble siRNA (Figure 7). Altogether, these data indicate that silencing Maclpil promotes anti-inflammatory macrophage polarization in association with increased fatty acid oxidation.

## Discussion

We previously showed increased expression of LncRNA Maclpil in hematopoietic cell-derived macrophages in the ischemic brain and the attenuation of ischemic stroke after transferring Maclpil-silencing macrophages (12). In this study, we demonstrated that systemically silencing Maclpil with siRNA reduced infarction size and brain inflammation in experimental ischemic stroke. In OGD experiments simulating *in vivo* ischemia, silencing Maclpil promoted macrophage apoptosis as well as anti-inflammatory macrophage activation *via* fatty acid oxidation.

Lymphocyte cytosolic protein 1 (LCP1) is L-plastin or leukocyte-specific plastin (17) targeted by lncRNA Maclpil (12). LCP1 plays a curtail role in immune responses by regulating cell adhesion, migration, and activation (12, 18, 19). LCP1 also affects cell fate. For example, LCP1 increased the resistance of cancer cells to apoptosis (20). In our previous study, knocking down lncRNA Maclpil downregulated LCP1 mRNA levels in macrophages (12). In OGD assay, siRNA silencing Maclpil accelerated apoptosis in macrophages by increasing the expression of pro-apoptotic genes Bax and Caspase3. Altogether, these results suggest that Maclpil promotes apoptosis in macrophages by modulating LCP1 expression.

Following ischemic stroke, leukocytes migrate from blood vessels into the inflamed brain with bone marrow-derived macrophages outnumbering other subsets of leukocytes (5, 21). In the acute phase, pro-inflammatory macrophages are dominant and promote inflammation in the ischemic hemisphere (22). In our previous study, knocking down lncRNA Maclpil promoted macrophage polarization toward anti-inflammatory macrophage phenotype as indicated by increased expression of Arg1 (12). In current OGD assays, siRNA silencing Maclpil enhanced the expression of anti-inflammatory macrophage markers as evidenced by elevated mRNA levels of Arg1, Ym1, and Fizz1 while attenuating the expression of proinflammatory macrophage markers such as iNOS, IL-1β, and TNF-α. Pro- and anti-inflammatory macrophages are metabolically distinct. Proinflammatory macrophages rely on aerobic glycolysis while anti-inflammatory macrophages use fatty acid oxidation to fuel mitochondrial oxidative phosphorylation (23–27). In addition, the lipid metabolism could affect the polarization of macrophages (28). Consistently, our data showed that the mRNA expression levels of 3 key enzymes in the fatty acid oxidation pathway were increased in macrophages in response to OGD after silencing Maclpil. These results suggest that enhancement of anti-inflammatory macrophage activation by inhibiting Maclpil under OGD conditions may be attributed to increased fatty acid oxidation.

Several studies have shed light on the role of LCP1, one of the lncRNA Maclpil target genes, in metabolic regulation. In a genome-wide association study (GWAS) (29), LCP1 was associated with non-alcoholic fatty liver disease. LCP1 polymorphism was also linked to reduced fasting insulin levels. Conversely, LCP1 deficiency augmented lipid catabolism (30). Additionally, macrophage shape also influences the activation status (31). Thus, it is reasonable to assume that the Maclpil-LCP1 axis may affect macrophage activation by regulating metabolism. However, further studies are warranted to define the role of LCP1 in macrophage metabolism and activation.

Several studies shed light on siRNA injection as a therapeutic treatment for stroke (32). For instance, Campbell et al. showed that the injected claudin-5 siRNA *via* the tail vein decreased cerebral edema in an animal models (33). In 2019, Kim et al. showed that intravenously delivery of high mobility group box-1 siRNA by exosomes was an effective therapy for ischemic stroke (34). In this study, our data showed that systemically siRNA silencing Maclpil by intravenously injected siRNA nanoparticles attenuated ischemic stroke, neurological defects, and brain inflammation in mice while promoting macrophage apoptosis and anti-inflammatory macrophage activation in response to OGD. Our study suggests that Maclpil or its regulated genes may serve as therapeutic targets for treating ischemic stroke.

## Data Availability Statement

The original contributions presented in the study are included in the article/supplementary material, further inquiries can be directed to the corresponding author.

## Ethics Statement

The animal study was reviewed and approved by Peking University and Capital Medical University Animal Care and Use Committees.

## Author Contributions

YW and BX contributed to the conception and design of the study. YW, CL, YC, and TH performed experiments, analyzed, and interpreted data. LX discussed the results together with YW. YW, TC, and BX wrote the manuscript. All authors read and approved the contents of the final manuscript.

## Conflict of Interest

The authors declare that the research was conducted in the absence of any commercial or financial relationships that could be construed as a potential conflict of interest.

## Publisher’s Note

All claims expressed in this article are solely those of the authors and do not necessarily represent those of their affiliated organizations, or those of the publisher, the editors and the reviewers. Any product that may be evaluated in this article, or claim that may be made by its manufacturer, is not guaranteed or endorsed by the publisher.

## Abbreviations

Abbreviates:

ACADM: Acyl-Coenzyme A dehydrogenase, C-4 to C-12 straight chain
ACADVL: ACAD, Very long-chain specific acyl-CoA dehydrogenase
HADHA: hydroxyacyl-CoA dehydrogenase/3-ketoacyl-CoA thiolase/enoyl-CoA hydratase
lncRNAs: Long non-coding RNAs
LCP1: Lymphocyte cytosolic protein 1
Maclpil: *Ma*crophage *c*ontained *L*CP1 related *p*ro-inflammatory lncRNA
MCAO: Middle cerebral artery occlusion
OGD: Oxygen glucose deprivation.

## References

[1] Hankey GJ. Secondary stroke prevention. *Lancet Neurol.* (2014) 13:178–94. 10.1016/S1474-4422(13)70255-2 24361114

[2] Kleindorfer DO Towfighi A Chaturvedi S Cockroft KM Gutierrez J Lombardi-Hill D 2021 Guideline for the prevention of stroke in patients with stroke and transient ischemic attack: a guideline from the American heart association/American stroke association. *Stroke.* (2021) 52:e364–467. 34024117 10.1161/STR.0000000000000375

[3] Li S Campbell BCV Schwamm LH Fisher M Parsons M Li H Tenecteplase reperfusion therapy in acute ischaemic cerebrovascular events-II (TRACE II): rationale and design. *Stroke Vasc Neurol.* (2021) 7:71–6. 10.1136/svn-2021-001074 34446531 PMC8899655

[4] Fu Y Liu Q Anrather J Shi FD. Immune interventions in stroke. *Nat Rev Neurol.* (2015) 11:524–35. 10.1038/nrneurol.2015.144 26303850 PMC4851339

[5] Li Y Wang Y Yao Y Griffiths BB Feng L Tao T Systematic study of the immune components after ischemic stroke using CyTOF techniques. *J Immunol Res.* (2020) 2020:9132410. 10.1155/2020/9132410 32908941 PMC7474762

[6] Zhang J Yuan L Zhang X Hamblin MH Zhu T Meng F Altered long non-coding RNA transcriptomic profiles in brain microvascular endothelium after cerebral ischemia. *Exp Neurol.* (2016) 277:162–70. 10.1016/j.expneurol.2015.12.014 26746985 PMC4761283

[7] He W Wei D Cai D Chen S Li S Chen W. Altered long non-coding RNA transcriptomic profiles in ischemic stroke. *Hum Gene Ther.* (2017) 29:719–32. 10.1089/hum.2017.064 29284304

[8] Dykstra-Aiello C Jickling GC Ander BP Shroff N Zhan X Liu D Altered expression of long noncoding RNAs in blood after ischemic stroke and proximity to putative stroke risk loci. *Stroke.* (2016) 47:2896–903. 10.1161/STROKEAHA.116.013869 27834745 PMC5127755

[9] Derrien T Johnson R Bussotti G Tanzer A Djebali S Tilgner H The GENCODE v7 catalog of human long noncoding RNAs: analysis of their gene structure, evolution, and expression. *Genome Res.* (2012) 22:1775–89. 10.1101/gr.132159.111 22955988 PMC3431493

[10] Lein ES Hawrylycz MJ Ao N Ayres M Bensinger A Bernard A Genome-wide atlas of gene expression in the adult mouse brain. *Nature.* (2007) 445:168–76. 17151600 10.1038/nature05453

[11] Mercer TR Dinger ME Sunkin SM Mehler MF Mattick JS. Specific expression of long noncoding RNAs in the mouse brain. *Proc Natl Acad Sci U S A.* (2008) 105:716–21. 10.1073/pnas.0706729105 18184812 PMC2206602

[12] Wang Y Luo Y Yao Y Ji Y Feng L Du F Silencing the lncRNA Maclpil in pro-inflammatory macrophages attenuates acute experimental ischemic stroke via LCP1 in mice. *J Cereb Blood Flow Metab.* (2020) 40:747–59. 10.1177/0271678X19836118 30895879 PMC7168792

[13] Wang Y Jin H Yao Y Yang C Meng J Tan X Sult2b1 deficiency exacerbates ischemic stroke by promoting pro-inflammatory macrophage polarization in mice. *Theranostics.* (2021) 11:10074–90. 10.7150/thno.61646 34815805 PMC8581421

[14] Schaar KL Brenneman MM Savitz SI. Functional assessments in the rodent stroke model. *Exp Transl Stroke Med.* (2010) 2:13. 10.1186/2040-7378-2-13 20642841 PMC2915950

[15] Schneider CA Rasband WS Eliceiri KW. NIH image to ImageJ: 25 years of image analysis. *Nat Methods.* (2012) 9:671–5. 10.1038/nmeth.2089 22930834 PMC5554542

[16] Liu Q Fan X Zhu J Xu G Li Y Liu X. Co-culturing improves the OGD-injured neuron repairing and NSCs differentiation via notch pathway activation. *Neurosci Lett.* (2014) 559:1–6. 10.1016/j.neulet.2013.11.027 24284009

[17] Morley SC. The actin-bundling protein L-plastin: a critical regulator of immune cell function. *Int J Cell Biol.* (2012) 2012:935173. 10.1155/2012/935173 22194750 PMC3238366

[18] Zeng Q Li L Feng Z Luo L Xiong J Jie Z LCP1 is a prognostic biomarker correlated with immune infiltrates in gastric cancer. *Cancer Biomark.* (2021) 30:105–25. 10.3233/CBM-200006 32986657 PMC12499957

[19] Shinomiya H Hirata H Saito S Yagisawa H Nakano M. Identification of the 65-kDa phosphoprotein in murine macrophages as a novel protein: homology with human L-plastin. *Biochem Biophys Res Commun.* (1994) 202:1631–8. 10.1006/bbrc.1994.2120 8060349

[20] Janji B Vallar L Al Tanoury Z Bernardin F Vetter G Schaffner-Reckinger E The actin filament cross-linker L-plastin confers resistance to TNF-alpha in MCF-7 breast cancer cells in a phosphorylation-dependent manner. *J Cell Mol Med.* (2010) 14:1264–75. 10.1111/j.1582-4934.2009.00918.x 19799649 PMC3828844

[21] Shi K Tian DC Li ZG Ducruet AF Lawton MT Shi FD. Global brain inflammation in stroke. *Lancet Neurol.* (2019) 18:1058–66. 10.1016/S1474-4422(19)30078-X 31296369

[22] Liu ZJ Ran YY Qie SY Gong WJ Gao FH Ding ZT Melatonin protects against ischemic stroke by modulating microglia/macrophage polarization toward anti-inflammatory phenotype through STAT3 pathway. *CNS Neurosci Ther.* (2019) 25:1353–62. 10.1111/cns.13261 31793209 PMC6887673

[23] Liu PS Ho PC. Determining macrophage polarization upon metabolic perturbation. *Methods Mol Biol.* (2019) 1862:173–86. 10.1007/978-1-4939-8769-6_13 30315468

[24] Nomura M Liu J Rovira II Gonzalez-Hurtado E Lee J Wolfgang MJ Fatty acid oxidation in macrophage polarization. *Nat Immunol.* (2016) 17:216–7. 10.1038/ni.3366 26882249 PMC6033271

[25] Yan J Horng T. Lipid metabolism in regulation of macrophage functions. *Trends Cell Biol.* (2020) 30:979–89. 10.1016/j.tcb.2020.09.006 33036870

[26] Remmerie A Scott CL. Macrophages and lipid metabolism. *Cell Immunol.* (2018) 330:27–42. 10.1016/j.cellimm.2018.01.020 29429624 PMC6108423

[27] Galván-Peña S O’Neill LA. Metabolic reprograming in macrophage polarization. *Front Immunol.* (2014) 5:420. 10.3389/fimmu.2014.00420 25228902 PMC4151090

[28] Wu HM Ni XX Xu QY Wang Q Li XY Hua J. Regulation of lipid-induced macrophage polarization through modulating peroxisome proliferator-activated receptor-gamma activity affects hepatic lipid metabolism via a Toll-like receptor 4/NF-κB signaling pathway. *J Gastroenterol Hepatol.* (2020) 35:1998–2008. 10.1111/jgh.15025 32128893

[29] Adams LA White SW Marsh JA Lye SJ Connor KL Maganga R Association between liver-specific gene polymorphisms and their expression levels with nonalcoholic fatty liver disease. *Hepatology.* (2013) 57:590–600. 10.1002/hep.26184 23213074

[30] Subramani M Yun JW. Loss of lymphocyte cytosolic protein 1 (LCP1) induces browning in 3T3-L1 adipocytes via β3-AR and the ERK-independent signaling pathway. *Int J Biochem Cell Biol.* (2021) 138:106053. 10.1016/j.biocel.2021.106053 34371171

[31] McWhorter FY Wang T Nguyen P Chung T Liu WF. Modulation of macrophage phenotype by cell shape. *Proc Natl Acad Sci U S A.* (2013) 110:17253–8. 10.1073/pnas.1308887110 24101477 PMC3808615

[32] Fukuda AM Badaut J. siRNA treatment: “A Sword-in-the-Stone” for acute brain injuries. *Genes (Basel).* (2013) 4:435–56. 10.3390/genes4030435 24705212 PMC3924829

[33] Campbell M Hanrahan F Gobbo OL Kelly ME Kiang AS Humphries MM Targeted suppression of claudin-5 decreases cerebral oedema and improves cognitive outcome following traumatic brain injury. *Nat Commun.* (2012) 3:849. 10.1038/ncomms1852 22617289

[34] Kim M Kim G Hwang DW Lee M. Delivery of high mobility group box-1 siRNA using brain-targeting exosomes for ischemic stroke therapy. *J Biomed Nanotechnol.* (2019) 15:2401–12. 10.1166/jbn.2019.2866 31748020

